# A Rare Case of an Infant With 1p36 Deletion Syndrome Presenting With Systolic Heart Failure Secondary to Severe Dilated Cardiomyopathy

**DOI:** 10.7759/cureus.45746

**Published:** 2023-09-21

**Authors:** Chukwunonye O Ogbuji, Lucio E Ortega, Haven Ward, Nzubechukwu Ugochukwu, Rakesh Donthula, Srilatha Alapati

**Affiliations:** 1 Pediatrics, Texas Tech University Health Sciences Center, Amarillo, USA; 2 Pediatric Cardiology, Rady Children's Hospital, San Diego, USA; 3 General Surgery, AdventHealth Florida Hospital, Orlando, USA; 4 Internal Medicine, Nnamdi Azikiwe University Teaching Hospital, Nnewi, NGA

**Keywords:** 1p36 deletion syndrome, genetic, left ventricular non-compaction cardiomyopathy, dilated cardiomyopthy, heart failure

## Abstract

1p36 deletion syndrome is a common terminal chromosomal deletion syndrome in humans. It is caused by the deletion of genetic material from a specific region in the short arm of chromosome 1. Symptoms range from seizure disorders, abnormalities of tone, visual and auditory disturbances. Cardiac abnormalities like left ventricular non-compaction (LVNC) and dilated cardiomyopathies (DCM) are commonly associated with this syndrome. This case report presents a 15-month-old female with dilated cardiomyopathy associated with 1p36 deletion syndrome, who has been followed from birth. Cardiac function was normal at birth with an ejection fraction of 65%. At three weeks of age, the patient presented with severe tachypnea, cyanosis, poor weight gain, and diaphoresis with feeding. Echocardiogram showed an ejection fraction of 22%. The patient was diagnosed with Modified Ross Heart Failure Class III. The patient was admitted to the cardiovascular intensive care unit where diuretics, phosphodiesterase inhibitors, and ionotropic agents were used to manage the heart failure. The patient relapsed two months later following a severe adenovirus infection. She was readmitted and heart failure medications were optimized. This patient has maintained a steady growth, meeting most milestones with no further relapse. The heterogeneity of 1p36 deletion syndrome presentation poses a diagnostic challenge for most clinicians. Cardiac involvements are very common and infants presenting with signs and symptoms of heart failure need to be screened for chromosomal abnormalities when other causes have been ruled out.

## Introduction

1p36 deletion syndrome is a chromosome disorder caused by the deletion of genetic material from a specific region in the short arm of chromosome 1. Only about 20% of cases are inherited from an unaffected parent with a balanced translocation [1]. It is associated with severe intellectual disability, some individuals with this deletion syndrome are unable to speak or have a few words in their vocabulary. Many of these individuals have structural abnormalities of the brain predisposing them to seizures, abnormalities of tone, and difficulties with swallowing. Heads are usually small with visual and auditory impairments, abnormalities of the skeletal, gastrointestinal, genitourinary, and cardiovascular systems also occur in these individuals [1].

High-risk cardiac abnormalities have been linked to this terminal deletion, these include congenital heart diseases (CHDs), cardiomyopathies (CMP), and potential progressive aortic dilatation [2]. Atrial septal defects (ASD), ventricular septal defects (VSD), patent ductus arteriosus (PDA), valvular anomalies, Coarctation of the Aorta (CoA), and Tetralogy of Fallot (TOF) are some of the commonest CHDs associated with 1p36 deletion syndrome [2]. While most of the cardiomyopathies noted with 1p36 deletion syndrome are left ventricular non-compaction (LVNC), severe dilated cardiomyopathies are less frequently seen in these patients [2].

LVNC cardiomyopathy is a heart defect in which the left ventricle has prominent trabeculations and deep inter-trabecular recesses [3]. LVNC can lead to heart failure, arrhythmias, cerebrovascular accidents, or even sudden death [3]. Most individuals with 1p36 deletion syndrome have LVNC while fewer cases of dilated cardiomyopathy have been demonstrated with 1p36 deletion [4]. In a large (60 patients) cohort study by Battaglia et al., 71% of patients had cardiovascular malformations, and 27% had cardiomyopathy. Among individuals with cardiomyopathy, 85% had LVNC and 15% had dilated cardiomyopathy [4]. This article highlights a rare case of 1p36 deletion syndrome presenting with dilated cardiomyopathy.

## Case presentation

**Figure 1:**
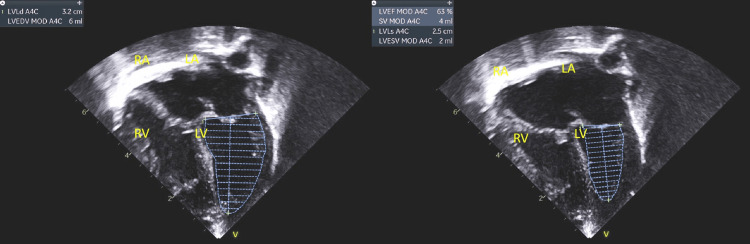
Apical 4 chamber view showing normal size LV with LVEF 65% done on 2nd day of life. LVEF: Left ventricle ejection fraction

The patient was conceived during maternal Tretinoin use, prenatal ultrasound showed ventriculomegaly and possible inferior vermian agenesis. Prenatal screens were negative for aneuploidies of 21, 18, and 13, and monosomies or trisomies of X/Y chromosomes. A 2-cord vessel was noted on delivery at term. On the 2nd day of life, an echocardiogram was done to evaluate a 3/6 systolic murmur. This revealed a moderate patent ductus arteriosus with low-velocity bidirectional shunting, both ventricles were normal in size and function (Figure 1). Ultrasound of the head showed large bilateral subependymal cysts. The newborn nursery course was uncomplicated.

**Figure 2:**
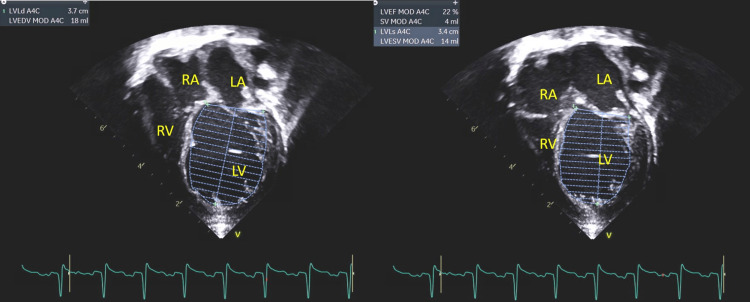
Apical 4 Chamber view showing severely dilated LV with LVEF 22% done at three weeks of age. LVEF: Left ventricle ejection fraction

**Figure 3:**
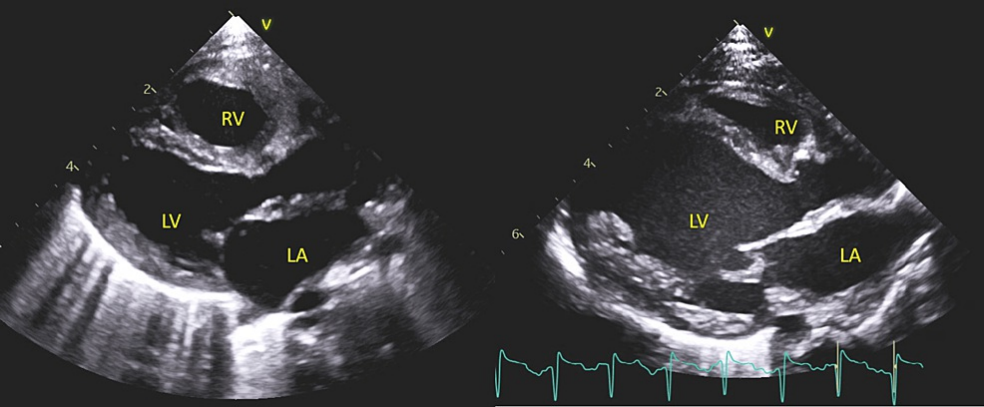
Parasternal long axis view showing the difference in LV size (left of screen done at DOL 2 – normal size) (right of the screen done at DOL 20 – severely dilated LV) DOL: Days of life

**Figure 4:**
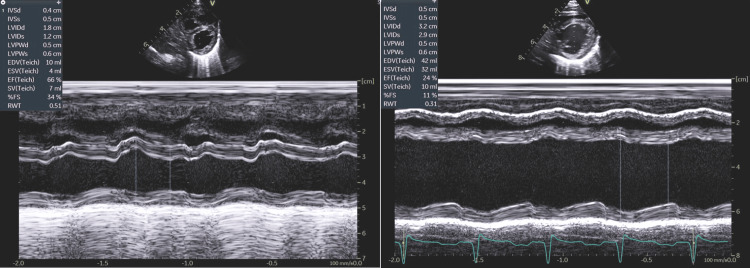
M-mode showing the difference at DOL 2 and DOL 20 DOL: Days of life

At three weeks of age, the patient presented to the emergency department with increased sleepiness, cyanosis, difficulty feeding, emesis, and poor weight gain. Physical examination showed a gallop rhythm and a repeat echocardiogram showed severely diminished left ventricular function with an ejection fraction (EF) of 20% (Figure 2). Figure 3 and Figure 4 make a comparison of echocardiographic findings on days of life (DOL) 2 and 20. The patient was admitted to the cardiovascular intensive care unit where Milrinone infusion, diuretics (Lasix & Diuril), Digoxin, and high-flow oxygen were started. Ivabradine was subsequently introduced for rate control on the 4th day of admission. A repeat echocardiogram on the 10th day of admission showed improved left ventricular function with an ejection fraction (EF) of 33%. Milrinone was weaned off and the dose of Captopril increased. Around day 13 of admission, Carvedilol was commenced, Captopril was transitioned to Enalapril, and Digoxin was weaned off. The patient convalesced and was transitioned to the acute care cardiology unit for further management of Modified Ross Heart Failure Class III where Ivabradine was discontinued and well tolerated. The patient was transitioned to oral heart failure medications (Lasix, Enalapril, and Carvedilol), maintained a steady weight gain on a 27Kcal per oz formula, and was discharged after 28 days on admission.

At 12 weeks of age, the patient had a worsening of her heart failure following a two-day history of nasal congestion, increased work of breathing, desaturations, and respiratory distress in the setting of adenovirus infection. An echocardiogram done at the emergency room showed a worsening ejection fraction of 18-20% from her baseline of 30-33%. She was admitted to the cardiovascular intensive care unit on intravenous Lasix with optimization of her home heart failure medications (enalapril and carvedilol). Cardiac function improved prior to discharge. At the time, she was noted to be in Modified Ross Heart Failure Class II.

On subsequent cardiology follow-ups, the patient’s ejection fraction has stayed at baseline (29-37%). The patient is currently 15 months old, and meeting most of her milestones with supportive therapy. She continues to take Lasix, Enalapril, Carvedilol, and Aspirin with no worsening of her condition.

## Discussion

1p36 deletion syndrome is the most common terminal chromosomal deletion syndrome in humans, occurring in 1 in 5000 newborns [5]. While specific loss of function gene mutations has been shown to cause some malformation, other genomic regions and dosage-sensitive genes that contribute to cardiac phenotypes caused by 1p36 deletions have not been clearly defined [4]. Defining the cardiac-related regions and genes on 1p36 will allow physicians to provide better medical care and faster diagnosis in pediatric patients with rare cardiac defects [4].

A publication by Jordan et al. suggested that efforts have been made to determine the smallest terminal deletion required for phenotypic expression [5]. Some critical regions have been identified with distinctive features of 1p36 deletion syndrome. Five critical regions along the entire length of the chromosome were defined by Zaveri et al. known to cause congenital heart defects [6]. Only two non-overlapping critical regions were identified for cardiomyopathies. The first is a distal critical region for LVNC while deletion of the proximal critical region was identified by Kang et al. in an individual with dilated cardiomyopathy [7].

Dilated cardiomyopathy is a disorder of the heart muscle manifesting with systolic dysfunction and dilatation of the left ventricle with normal left ventricular wall thickness. Some cases present with biventricular involvement [8]. About 50% of the cases are genetically determined with an autosomal pattern of inheritance [8]. Affected patients present with symptoms of heart failure - progressive dyspnea with exertion, impaired exercise capacity, orthopnea, paroxysmal nocturnal dyspnea, and peripheral edema - and these symptoms are most common in older children while infants can present with feeding intolerance (dyspnea and diaphoresis with feeding), cyanosis, and poor weight gain. Some cases are diagnosed by incidental detection of asymptomatic cardiomegaly and symptoms related to coexisting arrhythmia, conduction disturbance, thromboembolic complications, or sudden death [9].

The clinical and genetic heterogeneity observed among people with 1p36 deletions presents a significant challenge for clinicians who must provide prognostic information to families and generate individualized care plans for their patients [9]. The challenge arises, in part, because the genes that contribute to most 1p36-related phenotypes have not yet been fully identified, and many 1p36-related phenotypes may arise from the haploinsufficiency of more than one gene within a particular genomic region [9].

Data obtained from the Pediatric Cardiomyopathy Registry shows that the five-year rates for the combined outcome of death and transplant were 6% for LVNC and 43% for dilated cardiomyopathy [10].

## Conclusions

Systolic heart failure secondary to dilated cardiomyopathy associated with 1p36 gene deletion is an extremely rare form of cardiomyopathy. Infants presenting with signs and symptoms of heart failure should be screened for chromosomal abnormalities when other causes have been ruled out. This case highlights the phenotypic variability that occurs in patients with 1p36 deletions and the need for a better understanding of the phenotypic variations of the deletions.
